# Sample Delivery Media for Serial Crystallography

**DOI:** 10.3390/ijms20051094

**Published:** 2019-03-04

**Authors:** Ki Hyun Nam

**Affiliations:** 1 Division of Biotechnology, College of Life Sciences and Biotechnology, Korea University, Seoul 02841, Korea; structures@korea.ac.kr; Tel.: +82-10-5208-5730; 2 Institute of Life Science and Natural Resources, Korea University, Seoul 02841, Korea

**Keywords:** serial crystallography (SX), serial femtosecond crystallography (SFX), serial millisecond crystallography (SMX), X-ray free electron laser (XFEL), sample delivery, delivery medium, carrier matrix, viscous medium

## Abstract

X-ray crystallographic methods can be used to visualize macromolecules at high resolution. This provides an understanding of molecular mechanisms and an insight into drug development and rational engineering of enzymes used in the industry. Although conventional synchrotron-based X-ray crystallography remains a powerful tool for understanding molecular function, it has experimental limitations, including radiation damage, cryogenic temperature, and static structural information. Serial femtosecond crystallography (SFX) using X-ray free electron laser (XFEL) and serial millisecond crystallography (SMX) using synchrotron X-ray have recently gained attention as research methods for visualizing macromolecules at room temperature without causing or reducing radiation damage, respectively. These techniques provide more biologically relevant structures than traditional X-ray crystallography at cryogenic temperatures using a single crystal. Serial femtosecond crystallography techniques visualize the dynamics of macromolecules through time-resolved experiments. In serial crystallography (SX), one of the most important aspects is the delivery of crystal samples efficiently, reliably, and continuously to an X-ray interaction point. A viscous delivery medium, such as a carrier matrix, dramatically reduces sample consumption, contributing to the success of SX experiments. This review discusses the preparation and criteria for the selection and development of a sample delivery medium and its application for SX.

## 1. Introduction

The field of structural biology using synchrotron radiation has provided an understanding of the functional molecular mechanisms of macromolecules such as proteins and nucleic acids [1,2,3]. This has provided structural insights into the development of drug design for disease-related targets and the creation of improved enzyme activity for industry-applicable enzymes through rational engineering [3,4]. Despite this breakthrough technology, X-ray crystallography has several technical limitations, including radiation damage [5,6]. For example, a crystal sample is continuously exposed to X-rays during the full data collection at room temperature. During this process, *K*-shell photoionization and Auger decay in atoms occurs in the molecules, followed by electron cascade occurring by electron-impact ionization [7,8]. Thereafter, various radical reactions, with energies ranging between a few and several tens of electron volts, affect the molecules that differ in their timescales and temperature-dependence [9,10,11]. In particular, radiation damage can affect characteristics including redox processes, free radical generation, and chemical bond breakage, which represent irreversible changes in the electron density map [12,13,14]. Moreover, it can also affect the conformational changes of the molecules or crystal lattices [11]. As a result, radiation damage not only reduces the X-ray diffraction intensity of the crystal sample, but can also provide an electron density that often contains less reliable structural information. In order to minimize radiation damage, cryo-crystallography techniques are widely applied and dramatically reduce radiation damage [14,15,16]. However, this technique still involves radiation damage, as well as a cryogenic structure that may be biologically less reliable than the room temperature structure [14,17]. In particular, room-temperature structure determination is important for the elucidation of protein dynamics, demonstrating an accurate conformational flexibility that is crucial for understanding molecular function [18,19,20]. Moreover, room-temperature crystallography allows for time-resolved studies of protein dynamics and enzyme catalysis [21,22]. Therefore, macromolecule structure determination using a synchrotron X-ray source is experimentally limited for the collection of data with radiation damage in low temperature environments.

An X-ray free electron laser (XFEL) provides femtosecond X-ray pulses with an extremely high peak of brilliance [23,24,25,26,27]. The femtosecond pulse duration of the XFELs generates diffraction or scattering from the experiment sample, which is more rapid than their destruction by radiation damage [7,28,29], resulting in a crystal structure that does not include radiation damaged information. In serial femtosecond crystallography (SFX) using XFEL, the crystal samples are serially delivered to the X-ray interaction point at room temperature, and each crystal is exposed to X-rays only once [26,30]. As a result, SFX allows for the visualization of the crystal structure of macromolecules at room temperature without causing radiation damage [26,30]. Moreover, by using short-pulse X-rays, the dynamics of molecules that cause structural changes in a short period of time, such as photoactive proteins, can be observed [30,31,32]. Currently, there are five hard X-ray XFEL sources available for SFX: Linac Coherent Light Source (LCLS) [33], SPring-8 Angstrom Compact Free-Electron Laser (SACLA) [34], Pohang Accelerator Laboratory (PAL-XFEL) [35,36], European XFEL [37], and SwissFEL [38]. Meanwhile, the synchrotron microfocus beamline is also capable of collecting SX data at room temperature [20,39]. This is called serial millisecond crystallography (SMX) because of the long exposure times required in a synchrotron [39], which can reduce the radiation damage when compared to conventional X-ray crystallography using a single crystal.

In the first high-resolution SFX experiment, lysozyme crystals were delivered by a liquid jet sample injector with a gas dynamic virtual nozzle (GDVN) to an X-ray interaction point [40], and was used to successfully determine the crystal structure of lysozymes at 1.9 Å resolution [41]. However, the minimum flow rate and linear velocity of the GDVN-based injector was 10 μL/min and 10 m/s, respectively, which is too fast for XFELs, with a pulse repetition rate of less than 120 Hz [42]. Only one of tens of thousands of crystal samples will be hit by the X-ray pulse, and the rest of the sample will therefore be wasted [42]. As a result, the determination of the SFX structure using a liquid jet generally requires tens to hundreds of milligrams of protein [41,42]. Therefore, SFX studies using liquid jet injectors at low X-ray repetition rates are challenging due to the large amounts of crystal sample consumption. To overcome this issue, other sample delivery methods, such as electrospinning [43], LCP (lipidic cubic phase) injectors [42,44], acoustic injectors for drop-on-demand [45], or fixed-target scanning [46,47,48,49,50], have been developed to deliver crystal samples serially to X-ray interaction points in SX experiments [51].

**Figure 1:**
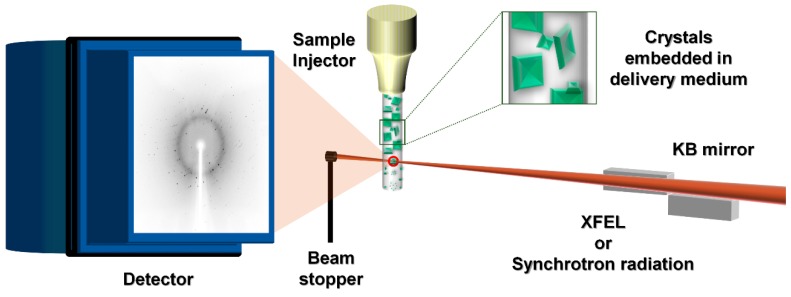
Schematic diagram of experimental geometry for serial crystallography using sample delivery medium. XFEL (X-ray free electron laser) or the synchrotron X-ray is focused using Kirkpatrick-Baez (KB) mirrors. The injection stream of the delivery medium containing crystals is extruded from the sample injector into the X-ray interaction point (red circle). The single panel detector without a center hole is the required beam stopper. Diffraction data is recorded by the detector.

Among them, the LCP microextrusion injector delivers the streaming of LCP using monoacylglycerol (MAG; e.g., monoolein) with embedded crystals at very slow flow rates (0.001–0.3 µL/min), which consumes 100–1000 times less sample than the liquid jet sample injector [41,42]. The flow rate of the crystal samples using the LCP injection can be fine-tuned against the XFEL’s pulse repetition rate, and the interval between pulses is long enough to avoid the damaged material out of the beam path [41,42]. After the development of LCP injection medium, other sample delivery media such as mineral-oil based grease [52], Vaseline (petroleum jelly) [53], agarose [54], hyaluronic acid (HA) [55], synthetic grease [55], hydroxyethyl cellulose (HEC) [56], nuclear grade grease [56], carboxymethyl cellulose sodium salt (NaCMC) [57], Pluronic F-127 (F-127) [57], poly(ethylene oxide) (PEO) [58], and polyacrylamide (PAM) [59] as the carrier matrix have been applied in SFX or SMX experiments. These sample delivery media commonly reduce the flow rate of the crystal samples from the sample injector and dramatically reduce sample consumption (Figure 1).

In order to conduct SFX or SMX studies using a delivery medium, it is important to understand the characteristics of the developed delivery medium and its applications in SX. This review describes the method of crystal sample mixing using the delivery medium reported so far and summarizes the content applied to SX for each delivery medium. In addition, the criteria for the selection of delivery materials and the preparation of injection experiments, as well as the requirements for the development of delivery medium, are discussed here.

## 2. Sample Preparation for the Crystals in Delivery Medium

**Figure 2:**
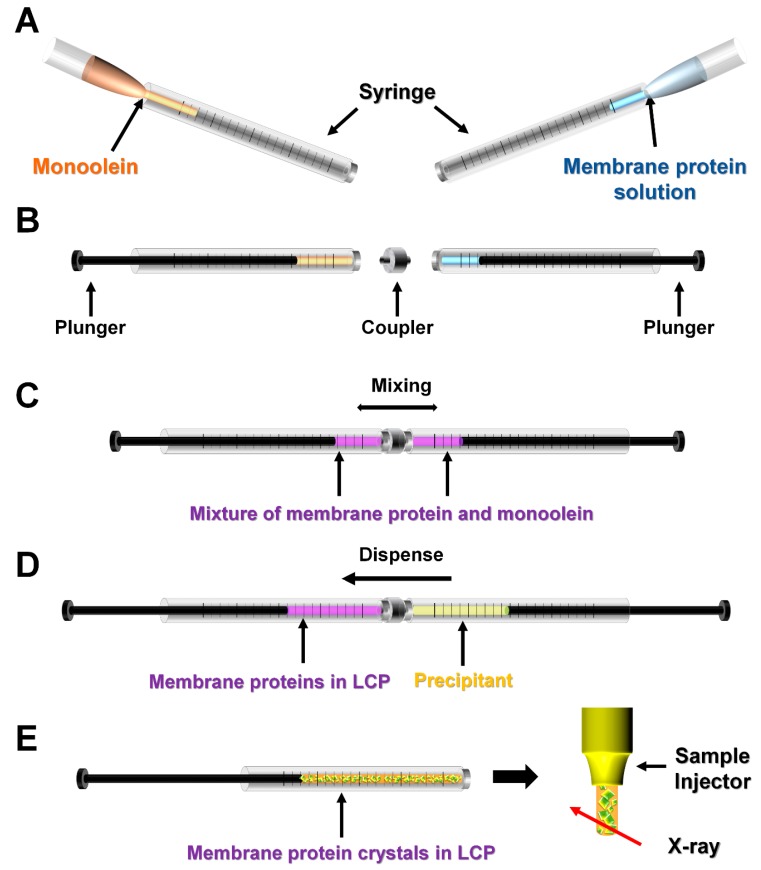
Crystal growth in delivery medium for serial crystallography. Example of crystallization of membrane protein in lipidic cubic phase (LCP). (**A**) Monoolein (9.9 MAG) as a delivery medium and membrane protein solution is injected into each syringe. The ratio of monoolein and protein solution is 3:2. (**B**) Syringes containing the monoolein and protein solution are connected using a coupler. (**C**) Mixing of membrane protein and monoolein forms LCP. (**D**) Crystallization solution is added to the syringe containing the mixture of membrane protein in LCP. (**E**) Crystal growth in LCP. After removing the precipitant, the LCP containing the crystals is transferred into the sample injector and is used to perform the serial crystallography (SX) experiment. This figure was drawn based on Reference [42]. A horizontal arrow indicates movement of the plunger. In vacuum, an additional titration step using short MAG is required (see text).

**Figure 3:**
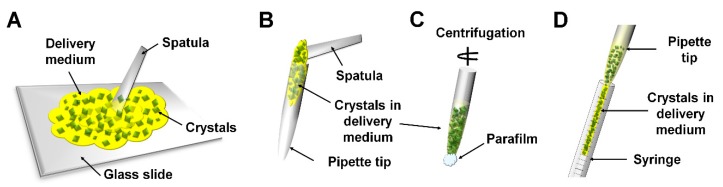
Schematic representation of manual mixing of crystals and delivery medium. (**A**) The crystals and the delivery medium are mixed using a spatula under a glass slide. (**B**) The mixture is transferred to the dispenser tip. (**C**) The mixture is then moved to the end of the tip using centrifugation. (**D**) The mixture is transferred to the syringe or sample injector. This figure was drawn based on Reference [52].

**Figure 4:**
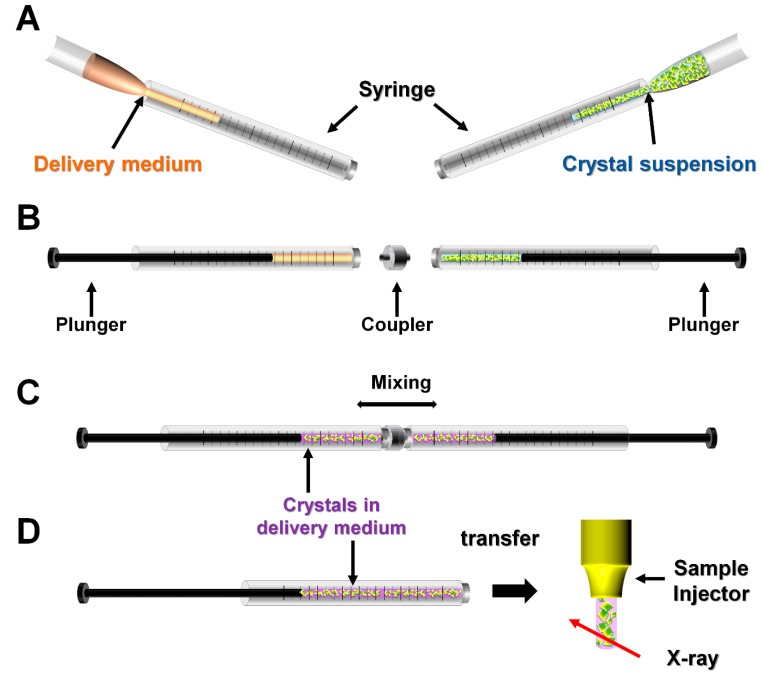
Schematic representation of mechanical mixing of crystals and delivery medium. (**A**) Delivery medium and crystal suspension are injected into separate syringes. (**B**) The syringes containing the delivery medium and the crystal suspension are connected using a coupler. (**C**) The crystal and delivery medium are gently mixed. (**D**) The delivery medium containing the crystals is transferred into a sample injector to perform the SX experiment.

In order to deliver crystal samples using a delivery medium for SX, the crystal samples must be embedded in the delivery medium. The following three methods for embedding the crystals in delivery medium have been reported so far: (i) crystal growth in delivery medium, (ii) manual mixing, and (iii) mechanical mixing (Figure 2, Figure 3 and Figure 4). When the crystal sample grows in a delivery medium that can be used as a delivery material, the SX experiment can be performed directly or after optimization for the stable and continuous injection of the sample from the sample injector. On the other hand, when crystallization is not available in the delivery medium, the crystallized sample is physically mixed with the delivery medium and transferred to the sample injector, after which the SX experiment is performed.

### 2.1. Crystal Growth in Delivery Media

This method is applicable when the protein is crystallized in delivery medium (Figure 2). This method was used for membrane protein crystals in an LCP (e.g., monoolein), which can be used as a delivery medium [60]. Lipidic cubic phase is an artificial membrane-mimicking gel-like material that forms spontaneously upon mixing of specific lipids and an aqueous solution [61]. Membrane proteins can be reconstituted into the lipid bilayer of LCP (Figure 2A–C). By adding the precipitant for crystallization (Figure 2D), the lipid/protein mixture undergoes crystal nucleation and growth [62] (Figure 2E). Since LCP is a viscous material suitable for use as a delivery medium, LCP-containing crystals can be directly used for SX experiments at ambient pressure after removing the crystallization solution (Figure 2E) [39]. Meanwhile, in vacuum, the sample delivery using LCP is required to mix shorter chain lipids (e.g., 9.7 MAG or 7.9 MAG) to avoid the transition of the LCP phase to the lamellar crystalline (Lc) phase (see below) [63]. On the other hand, previous SFX experiments attempted to crystallize phycocyanin (PC) from *Thermosynechococcus elongatus* in agarose gel for use as a delivery medium [54]. Due to its size and low diffusion constant, PC did not crystallize in agarose; however, since crystallization of other proteins in agarose gel has already been reported [64,65,66], it can be applied as an LCP in SX studies.

The advantage of the method of crystal growth in the delivery medium lies in its simple and convenient application to SX experiments. In addition, since this method has no mixing process between the crystal and the delivery medium, it results in little or no physical damage to the crystal sample when compared to other mixing methods. However, using this method, additional delivery optimization studies may be required depending on the property of the delivery material and the sample environment (e.g., LCP or agarose delivery medium in vacuum).

### 2.2. Manual Mixing

The manual mixing method involves directly mixing the crystal suspension and the delivery medium physically using a spatula (Figure 3). This method was used in the sample preparation process of various delivery media such as mineral-oil based grease [52], HA [55], synthetic grease [55], HEC [56], and nuclear grade grease [56]. For example, in the method using mineral oil grease, the grease spread on the glass slide, and crystal suspension was dispensed under the grease [52]. The grease and crystal suspension are manually mixed (Figure 3A) and placed into the dispenser tip using a spatula (Figure 3B). After sealing the exit port of the dispenser tip using Parafilm, the mixture was moved to the side of exit port in the dispenser tip by centrifuging for a few seconds (Figure 3C). Next, the grease containing the crystal samples in the dispenser tip was transferred to the sample injector using a pipette (Figure 3D). Then, the SFX experiment was performed [52]. The advantage of this method is simplicity and can be easily performed in the laboratory. Meanwhile, fragile crystal samples may be physically damaged during the manual mixing process. Rapid mixing may be required when a crystallization solution containing a chemical of high evaporation rate is included. Moreover, viscous materials may be deposited on the spatula or dispenser tip during the handling, which may result in sample loss.

### 2.3. Mechanical Mixing

The mechanical mixing method involves mixing the crystal suspension and the delivery medium physically using a syringe setup (Figure 4). This mixing method was originally developed for the crystallization of membrane proteins in LCP [67,68]. This method was applied using delivery mediums including LCP (for soluble proteins) [69], agarose [54], F-127 [57], PEO [70], and PAM [59]. For example, using agarose [54], the crystal suspension and the melted agarose were loaded into separate syringes (Figure 4A). These two syringes were connected using a coupler (Figure 4B), then mixed until the crystal suspension was uniformly distributed in the delivery medium (Figure 4C). This mixture was further transferred to the sample injector and used in the SFX experiment (Figure 4D). A similar procedure can also be applied for LCP (for soluble protein crystal) [69], F-127 [57], and PEO [70]. On the other hand, in the application of PAM, an additional step is required, in which the cross-linked PAM is disrupted to avoid the physical damage of crystal suspension during the syringe mixing by high strength PAM [59].

Compared to the manual mixing method, the mechanical mixing method using a syringe setup has the advantage of reduced sample loss and avoids the dehydration of the crystal sample. However, the crystal sample may be subjected to physical damage due to the use of a narrow coupler hole during the mixing process for the uniform distribution of crystals in the delivery medium.

## 3. Sample Delivery Media and its Applications

Based on the chemical properties, previously reported delivery media can be classified into three types, as follows: (i) LCP-based delivery medium; (ii) oil-based delivery medium: mineral oil grease [52], Vaseline [53], synthetic grease Super Lube [55], and nuclear grade grease [56]; (iii) hydrogel-based delivery medium: agarose [54], HA [55], HEC [56], NaCMC [57], F-127 [57], PEO [58], and PAM [59]. On the other hand, Mebio, guar, xanthan, guar, and tragacanth materials have also been suggested as possible delivery materials [53,57], but sufficient information has not been reported and these have been excluded from this review.

### 3.1. Lipidic Cubic Phase

**Figure 5:**
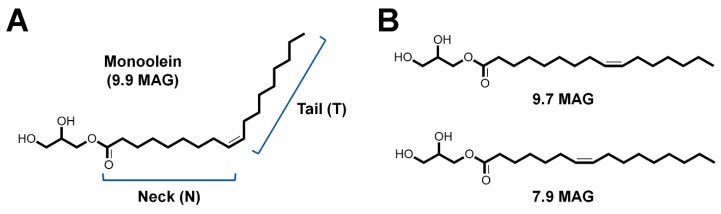
Chemical structure of monounsaturated monoacylglycerol lipids (MAGs). (**A**) Shorthand representation of N.T MAG, where N (neck) is the number of carbon atoms in the acyl chain between the ester and cis-olefin bonds and T (tail) is the number of carbon atoms between the cis-olefin bond and the end of the chain. (**B**) Chemical structure of short MAG lipid 9.7 MAG and 7.9 MAG, used in the SFX experiment in vacuum to avoid the Lc phase of 9.9 MAG.

Lipidic cubic phase (LCP) is a membrane-mimicking gel-like matrix for membrane protein crystallization in a lipidic environment [60]. Monoacylglycerols (MAGs) represent the most widely used lipid class for crystallization of membrane proteins by the LCP method [71]. Monoacylglycerols contain a glycerol head group and a hydrocarbon tail with a cis double bond (Figure 5). Most MAG lipids have very similar temperature-composition phase behavior [42]. Monoolein (9.9 MAG) is the most successful lipid used for LCP crystallization [72], although other MAGs have also been reported to be useful [73,74]. Although LCP using monoolein is useful for membrane protein crystallization, the LCP is cooled to the equilibrium phase transition temperature (~18 °C) by evaporation in vacuum, followed by the layering of a part of the sample into the lamellar crystalline phase (Lc). These patches of the Lc phase produce strong and sharp powder diffraction rings when exposed to an X-ray beam, which increase background scattering and may lead to damage of sensitive detectors [44]. This problem can be overcome by the addition of shorter chain lipids (9.7 MAG or 7.9 MAG) for lower phase transition temperatures. In contrast, LCP extrusion is performed at ambient pressure without the addition of shorter chain lipids [69]. The LCP microextrusion injector delivers a stable injection stream of LCP containing the crystal samples at a very slow flow rate of 0.001–0.3 μL/min, which varies depending on the sample composition, nozzle diameter, and pressure [44]. During the initial experiments, several G protein-coupled receptors, such as β_2_ adrenergic receptor, adenosine A_2A_ receptor, smoothened receptor (SMO), glucagon receptor, and serotonin 2B (5-HT2B) receptor, in LCP were stably delivered using the LCP injector with a flow rate of 170 nL/min [42,44]. Full data collection was performed using <0.5 mg of DgKA, SMO, and 5-HT2B [42,44]. The LCP shows the diffuse scattering and/or Debye–Scherrer rings at 4–5 Å. LCP provides a stable stream for most crystallization conditions; however, in some precipitants, the LCP phase can be transferred to the lamellar, hexagonal, or sponge phases [42], and is not compatible with high concentrations of ammonium sulfate [53]. LCP, on the other hand, is also used for delivering soluble protein crystals for reducing sample consumption [70].

### 3.2. Oil-Based Delivery Medium

#### 3.2.1. Mineral Oil Grease

The crystal suspensions were embedded in mineral oil grease using the manual mixing method with a spatula [52] (Figure 3). The mixture sample was derived using a syringe in a helium chamber [52]. The sample holder was exposed to a cooled helium gas stream and the microcrystals embedded in the grease were maintained at a temperature of 19.5–21.7 °C. The temperature and humidity in the sample chamber were 21.3–23.6 °C and 2–9%, respectively. A grease stream containing the lysozyme (crystal size: 7–10 µm in maximum length), glucose isomerase (10–30 µm), thaumatin (10–30 µm), and FABP3 (10–20 µm) crystals were extruded from a syringe injector using a 110 µm-ID (inner diameter) needle at a flow rate of 0.46–0.48 µL/min [52]. The crystal structures of lysozyme, glucose isomerase, thaumatin, and FABP3 were determined to be 2.0 Å, 2.0 Å, 2.0 Å, and 1.6 Å, respectively, using <1 mg of sample for all proteins [52]. The X-ray diffraction ring pattern and background diffraction from grease were observed at the ~14 Å and 4–5 Å regions, respectively [52].

#### 3.2.2. Vaseline (Petroleum Jelly)

For the SMX experiment, the crystal suspension was embedded in Vaseline using the mechanical mixing method with a syringe setup [53] (Figure 4). The mixture was delivered using a HVE (high viscosity extrusion) sample injector [53]. The flow speed of the Vaseline was in the range of 50 m/s to several mm/s, and the flow rate was 4–300 nL/min when a 40 μm-ID capillary was used [53]. Vaseline generated Debye–Scherrer rings at 4.2 and 3.77 Å spacing, and additional weak rings at higher resolutions [53]. The viscosity or flow rate of the Vaseline can be adjusted by adjusting the amount of crystallization solution during mixing [53]. Vaseline can form thinner streams during injection extrusion than LCP. Moreover, it is much silkier than LCP and is useful for embedding fragile crystals [53].

#### 3.2.3. Synthetic Grease Super Lube

For the SFX experiment, synthetic grease Super Lube was ground for 30–60 min using a mortar for use as a crystal delivery medium [55]. Ground synthetic grease Super Lube was mixed with crystal suspension using the manual method on a glass slide [55] (Figure 3). When the untreated synthetic grease Super Lube was delivered using a 110-μm ID nozzle, the grease was extruded to a thickness of ~210 μm, which is similar to the outer dimmer (OD) of the nozzle [55]. On the other hand, ground synthetic grease Super Lube grease extruded to a thickness of 110 μm. Since a thicker delivery medium increases the level of background scattering, ground synthetic grease Super Lube is more useful in SFX experiments. Super Lube grease shows a stronger background scattering in a resolution range of ~4.8 Å of all the diffraction images [55]. The crystals in synthetic grease Super Lube were kept at approximately 20 °C, and the temperature and humidity of the sample chamber were ~26 °C and >80%, respectively. Protein K (5–10 μm) and lysozyme (7–10 μm) crystals embedded in synthetic grease Super Lube were derived at a flow rate of 0.48 μL/min. The crystal structures of lysozyme and proteinase K embedded in synthetic grease Super Lube were both determined at a resolution of 2.3 Å [55].

#### 3.2.4. Nuclear Grease

In the SFX experiment, the salt-like impurities in the nuclease grease (Super Lube nuclear grade grease) were removed by filtration using a 10-μm mesh [56]. Lysozyme (5 × 5 × 5 μm) crystal suspensions were embedded in nuclease grease using manual mixing with a spatula (Figure 3). The nuclear grease matrix was extruded as a continuous stream with a diameter of ~100 μm through a 100-μm ID nozzle at a flow rate of 0.42 μL/min. The crystals in the nuclear grease were kept at approximately 20 °C in the injector, and the temperature and humidity of the sample chamber were ~26 °C and >50%, respectively. The crystal structure of the lysozyme delivered in the nuclear grease was determined at a resolution of 2.0 Å, using 0.5 mg of protein. Nuclear grease has a lower level of background scattering than other grease matrices, but higher than LCP [56].

### 3.3. Hydrogel-Based Delivery Medium

Hydrogels are three-dimensional hydrophilic polymer networks that are cross-linked through chemical or physical bonds [75]. They are capable of absorbing and retaining large quantities of water while maintaining their mechanical and physical form [75]. The hydrogel-based delivery media used in SX can be further divided into saccharides-based (agarose, HA, HEC, and NaCMC) and non-saccharide-based (F-127, PEO, and PAM) hydrogels. Saccharide-based delivery materials are considered potentially specific or non-specific interactors with sugar-related protein crystal samples.

#### 3.3.1. Agarose

**Figure 6:**
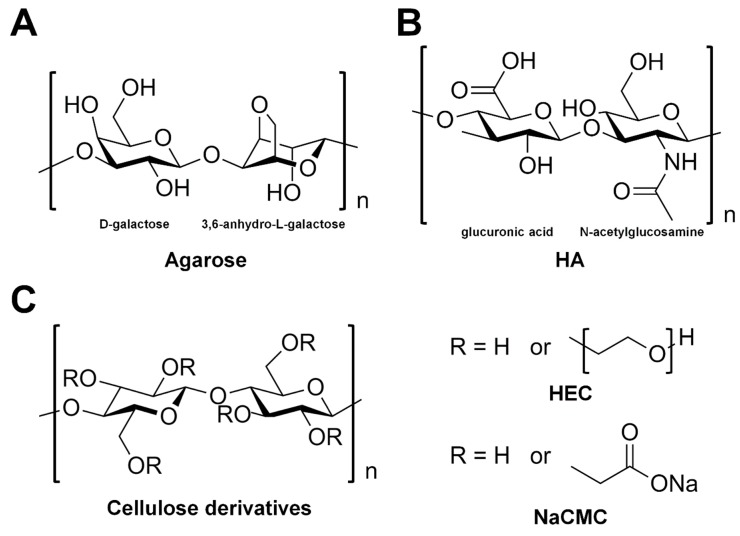
Chemical structure of polysaccharide-based hydrogels. (**A**) Agarose, composed of β-(1-4)-(3,6)-anhydro-l-galactose (left) and α-(1-3)-d-galactose (right). (**B**) Hyaluronic acid (HA) composed of alternating residues of β-d-(1-3) glucuronic acid (left) and β-d-(1-4)-*N*-acetylglucosamine (right). (**C**) Structure of cellulose derivatives. In hydroxyethyl cellulose (HEC), R is H or hydroxy ethyl group (–CH_2_CH_2_–OH). In carboxymethyl cellulose sodium salt (NaCMC), R is H or carboxymethyl groups (–CH_2_–COOH).

Agarose is a polysaccharide derived from seaweed, composed of a basic repeat unit consisting of alternating d-galactose and 3,6-anhydro-L-galactopyranose linked by α-(1→3) and β-(1→4) glycosidic bonds which undergo thermal crosslinking [76,77] (Figure 6A). In the SFX experiment, the crystal suspensions were mixed with agarose using the mechanical mixing method with a syringe setup [54] (Figure 4). During the initial experiment, the crystal samples embedded in agarose were derived to an X-ray interaction point in a vacuum at room temperature. However, the agarose medium was dehydrated and formed an ice formation under vacuum conditions [54]. To solve this problem, 30% (*v*/*v*) glycerol was added to the agarose media for cryoprotection in vacuum. After optimization of the stable sample injection, 5.6% (*w*/*v*) agarose dissolved in 30% (*v*/*v*) glycerol was selected, which formed a stable and continuous stream without ice-crystal diffraction. The agarose delivery medium can be used in an expansive temperature (4–30 °C). The phycocyanin (PC) crystals embedded in agarose were extruded from the LCP injector using a 50 µm-ID capillary into the X-ray interaction point at a flow rate of 160 nL/min. The room temperature structure of PC embedded in agarose was determined at a resolution of 2.5 Å using 0.3 mg of microcrystals. The diffuse X-ray background scattering of the agarose medium was observed in the region of 3.3 Å. The chemical compatibility of the agarose delivery medium for the crystallization of the precipitant demonstrates that the agarose injection stream was stable in 1 M NaCl, 1.25 M ammonium sulfate, 0.2 M NaCl, and >30% PEG 400–8000, and organic precipitants such as 2-methyl-2,4-pentanediol (MPD). Agarose can be used both in vacuum conditions and under ambient pressure. Stable stream conditions have been reported under vacuum conditions [54]; however, optimization conditions have not been reported under ambient pressure conditions.

#### 3.3.2. Hyaluronic Acid

Hyaluronic acid (HA) is widely distributed in the intercellular matrix of mammalian connective tissues, which are composed of a basic repeat unit consisting of alternating d-glucuronic acid and N-acetyl-d-glucosamine linked by β-(1→4) and β-(1→3) glycosidic bonds (Figure 6B) [78]. Although HA is a quite homogeneous polymer, the distributions of its molecular sizes are wide ranging (10^5^–10^7^) [78]. In the SFX experiment, the HA delivery medium was manually mixed with proteinase K (5–10 μm) and lysozyme (7–10 μm) crystals using a spatula on a glass slide [55] (Figure 3). Optimizing the HA solution buffer is required to prevent crystal damage. It is essential to mix the HA aqueous solution with the supernatant or crystal harvest solution before adding the crystal suspension, which avoids osmotic shock in the crystals when mixed with the medium [55]. A final concentration of ~12% (*w*/*v*) of HA was used to deliver the crystal suspension at an injection flow rate of 0.48 μm/min in a helium chamber. The crystal structures of proteinase K and lysozyme derived in HA were both determined at a resolution of 2.3 Å, using less than 1 mg of protein [55]. The background scattering peak appeared at a resolution of around 3.2 Å, which originated from the solvent. While HA has the advantage of providing a stable stream, this delivery material is costly compared with other delivery media [56].

#### 3.3.3. Hydroxyethyl Cellulose

Hydroxyethyl cellulose (HEC) is hydrophilic material derived from cellulose and contains a hydroxy ethyl group (–CH_2_CH_2_–OH) bound to the hydroxyl groups of the cellulose backbone [79] (Figure 6C). At concentrations below 5%, HEC (MW ~250,000) was not continuously extruded from the injector, and extrusion was difficult at ~30% HEC. A final concentration of 10–20% HEC was adequate to carry out the SFX experiment [56]. A final concentration of 16%, 11%, 22%, 16%, and 16% HEC medium were manually mixed with lysozyme (1 × 1 × 1 μm), lysozyme (20 × 20 × 30 μm), taumatin (2 × 2 × 4 μm), protein K (Pr soaking, 4 × 4 × 4–5 × 5 × 7 μm), and proteinase K (native 8 × 8 × 8–12 × 12 × 12 μm) crystals using a spatula on a glass slide (Figure 3). The microcrystals embedded HEC in the micro-extrusion injector were kept at a temperature of approximately 20 °C. The temperature and humidity in the sample chamber were ~26 °C and >50%, respectively. This delivery medium containing the crystals was delivered by sample injection at a flow rate of 0.38–0.75 μL/min. The crystal structure of the lysozyme and thaumatin delivered in HEC were determined at a resolution of 1.45 Å (and 1.8 Å for small crystals) and 1.55 Å, respectively. In this study, using HEC, de novo phasing in SFX by applying praseodymium-SAD (single-wavelength anomalous dispersion), single-isomorphous replacement (SIR), and SIR with anomalous scattering (SIRAS) phasing were demonstrated and used to determine the crystal structure of proteinase K at a resolution of 1.5 Å. The HEC medium provides a slightly higher level of background scattering over a resolution range of ~3.5–2.5 Å [56]. HEC has less adhesion than HA medium and prevents clogging of the sample catcher or adhesion of the injector nozzle surface [56]. In addition, HEC costs 1000 times less than HA, in terms of the price per gram [56].

#### 3.3.4. Carboxymethyl Cellulose Sodium Salt

Carboxymethyl cellulose (CMC) is derived from cellulose and contains carboxymethyl groups (–CH_2_–COOH) bound to the hydroxyl groups of the cellulose backbone [58] (Figure 6C). Carboxymethyl cellulose sodium salt (NaCMC) begins to form a rigid gel at 2.5%, and its viscosity is increased by increasing the NaCMC concentration; however, its solubility limit is slight above 10% [57]. A 7% (*w*/*v*) stock gel of NaCMC in water was heated at 60–70 °C and trapping of bubbles was minimized by homogenization with spatula [57]. Homogeneous thick gels were formed within 2 days at room temperature and stored at 4 °C to prevent contamination by microorganisms. A NaCMC delivery medium was obtained quickly by adding NaCMC slowly to vigorously stirred water in a beaker. Once all the powder was incorporated, the stirring speed was lowered and the mixture was heated under vacuum for 1 h at 60–70 °C. The NaCMC gel was then left under vacuum conditions overnight to fully hydrate and to remove any residual bubbles. This gel was mixed with crystal suspension using the mechanical mixing method with a syringe setup (Figure 4). The NaCMC delivery medium was then loaded with a 150-μm ID capillary at a flow rate of 0.3–5.9 μL/ min. The crystal structures of lysozyme and thermolysin derived from NaCMC were determined at resolutions of 1.9 Å and 2.3 Å, respectively, using <0.5 mg protein. This delivery medium has a very low background scattering with a weak diffuse ring at 2.6–4 Å. In terms of the chemical compatibility of NaCMC with crystallization precipitants, NaCMC provided a stable injection stream in high salt or PEGs crystallization solutions such as 1.25 M LiSO_4_, 1.25 M MgSO_4_, 1.8 M ammonium sulfate, 2 M sodium chloride, 35% (*w*/*v*) PEG 400, 30% (*w*/*v*) PEG 2000, 25% (*w*/*v*) PEG 4000, 25% (*w*/*v*) polypropylene glycol 400, and 35% (*v*/*v*) 2-methyl-2,4-pentanediol (MPD). As a result, NaCMC forms stable streams with a wide range of precipitants. However, medium preparation is time consuming compared with other delivery media. In addition, NaCMC is sensitive to crystal size, where the stability of the stream is disturbed when large crystals are embedded in NaCMC [57].

#### 3.3.5. Pluronic F-127

**Figure 7:**
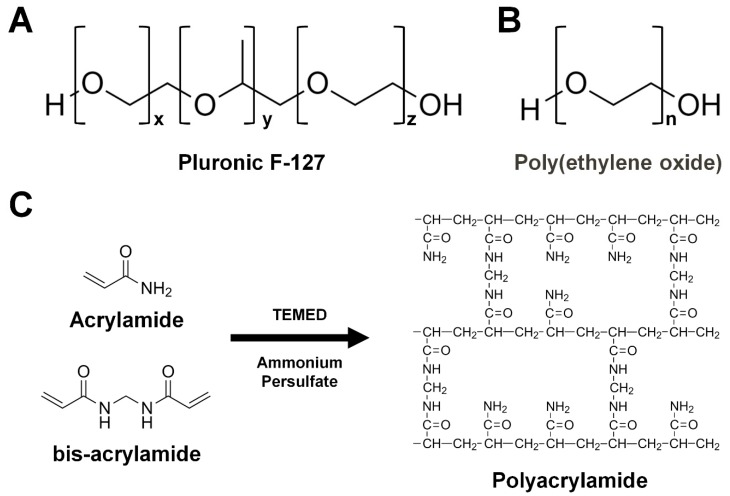
Chemical structure of polymer-based hydrogels. (**A**) Pluronic F-127. (**B**) Poly(ethylene oxide). (**C**) Polyacrylamide. Acrylamide and bis-acrylamide polymerize into polyacrylamide by adding the Tetramethylethylenediamine (TEMED) and ammonium sulfate.

Pluronic F-127 (F-127, Poloxamer 407, PF-127) is a non-ionic surfactant composed of polyoxyethylene-polyoxypropylene copolymers and is a thermoreversible gel [80,81] (Figure 7A). It forms monomolecular micelles at low concentrations (10^−4^–10^−5^%) and multimolecular aggregates consisting of a hydrophobic central core with hydrophilic polyoxyethylene chains facing the external medium at high concentrations [80,82] and 20–35% (*w*/*v*) F-127 forms a thermoreversible gel, which exists as a liquid at 4 °C and as a solid at room temperature [57]. F-127 was dissolved gently since it easily forms foam, and the pelleted/clumped F-127 polymer was gentle stirred 1–2 times a day in the a cool room until forming a gelling liquid [57]. The process of obtaining a clear viscous liquid of F-127 usually took three days. Crystal samples such as thermolysin, glucose isomerase, lysozyme, and bacteriorhodopsin in LCP crystals were mechanically mixed with cold 35% (*w*/*v*) F-127 stock solution using a syringe setup (Figure 4). Crystal samples embedded in F-127 were derived using an HVE injector with a 100-μm ID capillary. The crystal structures of glucose isomerase, thermolysin, and bacteriorhodopsin-LCP derived from F-127 were determined at resolutions of 2.0 Å, 2.0 Å, and 2.3 Å, respectively, using less than 0.5 mg of protein for each dataset [57]. The diffuse scattering of F-127 was observed at around 2.8–5 Å, with a scattering intensity level similar to that of grease, and wider and stronger than that of other hydrogels [57]. In terms of chemical compatibility, the injection stability of F-127 tolerates only NaCl and low-molecular weight PEG/PPG. The injection medium of F-127 was stable at a final concentration of 0.25 M ammonium sulfate, 2 M NaCl, 25% (*w*/*v*) polypropylene glycol 400, 23% (*w*/*v*) polyethylene glycol 400, and 7% (*w*/*v*) polyethylene glycol 2000 [57]. F-127 preparation was time consuming compared to other delivery media.

#### 3.3.6. Poly(ethylene oxide)

Poly(ethylene oxide) (PEO) is a water-soluble synthetic polymer and has the same chemical composition as PEG (polyethylene glycol), but a larger molecular weight (Figure 7B) [58]. This material is often used in crystallization as precipitant. Poly(ethylene oxide) (MW ~8,000,000) powder was dissolved in a syringe by mechanical mixing using a syringe setup [58]. After removing air and dissolving the PEO, the crystal suspension was embedded in the PEO gel uniformly by mechanical mixing using a syringe setup (Figure 4). The phycocyanin (~20 μm), Flpp3 in LCP (~20 μm), and proteinase K (10–15 μm) crystals enclosed in PEO were delivered at an average flow rate of 182 nL/min (crystal velocity: 1550 μm/s), 155 nL/min (1315 μm/s), and 79 nL/min (675 μm/s), respectively. The room temperature crystal structures of phycocyanin, Flpp3, and proteinase K were determined at 3.1, 3.0, and 2.65 Å, respectively. Poly(ethylene oxide) showed diffuse scattering at a resolution of around 3.3 Å, which mostly consisted of water scattering. The sample preparation using PEO gel as the delivery medium was simple and straightforward and was highly stable at a wide range of temperatures, including the traditional crystallization temperatures of 4–30 °C [58]. The PEO gel was compatible with a wide variety of precipitants commonly used in protein crystallization, including organic solvents [58].

#### 3.3.7. Polyacrylamide

Polyacrylamide (PAM) is formed from acrylamide subunits and is a non-toxic polymer (–CH_2_CHCONH_2_–) (Figure 7C) [83]. Cross-linked PAM forms a soft gel in the presence of water and exhibits stability at a wide pH range (pH 3–11) [84,85]. Polyacrylamide does not specifically or non-specifically interact with proteins [86]. Therefore, the crystal samples can be stored stably in PAM delivery materials. In the SFX experiment, the PAM solution was loaded into the syringe and then left to stand until it was thermally removed during the polymerization process [59]. To avoid physically damaging the crystals during mixing with high strength PAM, cross-linked PAM was disrupted into PAM fragments using a dual-syringe setup [59]. Crystal suspensions were embedded in PAM fragments by mechanical mixing using a syringe setup (Figure 4). The 10% (*w*/*v*) PAM provided a stable stream at a flow rate of 800 nL–2 μm/min at room temperature in either an air or helium chamber [59]. However, at a flow rate < 400 nL/min, Debye–Scherrer rings were observed at ~4 Å and dehydration occurred as a result of using helium gas in the outer capillary of the injector [59]. The crystal structures of lysozyme and thermolysin derived in PAM were determined at 1.7 and 1.8 Å, respectively, using 0.5 mg lysozyme and 1 mg thermolysin. The diffuse scattering from PAM was observed near a resolution of 3.2 Å, and mostly consisted of solvent scattering. In terms of its chemical compatibility with crystallization precipitants, 10% (*w*/*v*) PAM delivered a stable stream in high salts such as 2 M (NH_4_)_2_SO_4_ and 2.1 M NaCl. In contrast, 15% (*w*/*v*) PAM delivered a stable stream only at low concentrations in polymer or organic solvents such as 10% (*w*/*v*) PEG 400, 7% (*w*/*v*) PEG 1000, 5% (*w*/*v*) PEG 4000, 5% (*w*/*v*) PEG 8000, or 10% (*v*/*v*) MPD. Polyacrylamide was found to be suitable when using high salt precipitants; however, it showed a decrease in viscosity under high concentration polymers or organic solvent with an unstable stream [59].

## 4. Discussion

In this review, the experimental preparation and application of delivery media for SX experiments were described. The delivery media dramatically reduced the consumption of crystal samples by lowering the flow rate from the sample injector using the properties of highly viscous materials. In SFX experiments using delivery materials, all the crystal structures were determined using less than 1 mg of protein. This low amount of protein consumption facilitates the SFX research approach and provides an opportunity to develop new research. Although the reported delivery media were demonstrated to be success for SX applications, the optimization of sample delivery media for stable injection is required according to the experimental conditions of each facility (X-ray source, temperature, vacuum or ambient pressure, humidity). Moreover, when conducting SX studies using a delivery medium, preliminary studies are essential to ensure the stability between the crystal sample and the delivery medium, as well as a stable injection stream of the delivery medium containing the crystals from the sample injector. The following criteria should be taken into account when selecting a delivery material: (i) the delivery material should not undergo any chemical reaction with the sample crystals; (ii) the viscosity of the delivery medium for a stable flow rate should not be affected by the crystallization solution; (iii) the delivery medium should not damage the crystal during sample mixing; (iv) under vacuum conditions, when the cryoprotectant solution is added to the delivery medium to prevent dehydration by cooled evaporation, the crystal sample should not be affected. After selecting the delivery medium to be used for the SX experiment, whether the crystal sample is stable without chemical reaction when mixed with the delivery medium and whether the crystal sample is damaged according to the physical mixing method should be confirmed. To confirm the stability of the crystals in the selected delivery medium, high-resolution microscopy can be used to determine whether the crystal morphology has changed or dissolved. Second order non-linear imaging of chiral crystals (SONICC), including second harmonic generation (SHG) and ultraviolet two-photon excited fluorescence (UV-TPEF) can also be used to easily visualize the protein crystal sample in the delivery medium [87,88]. The most accurate evaluation of the stability of the crystals in the delivery medium involves measuring the diffraction intensity by exposing the crystals to X-rays after long-term incubation in the delivery medium and then comparing them to the diffraction intensity of the native crystals. This experiment can be performed using X-rays from a synchrotron or home source.

Next, the selected delivery medium can vary in viscosity depending on the crystallization solution, which is related to the stability of the stream delivered from the injector. A preliminary study of the viscosity of the delivery material for crystal solutions is essential for the efficient use of beamtime at XFELs or synchrotrons. Agarose [54], NaCMC [57], F-127 [57], and PAM [59] have been studied through chemical compatibility tests for precipitants in crystallization solution. This information can be used as a guide for selecting the delivery medium in further research. In contrast, in the case of other delivery media, chemical compatibility with various precipitants in the crystallization solution was not fully performed or not described in detail. Additional chemical compatibility studies on the crystallization solution would be required to provide information on the delivery material selection. As in previous studies of chemical compatibility in NaCMC [57], F-127 [57], and PAM [59], the delivery medium containing crystals can be manually ejected from a syringe and the stability of the delivery medium stream can then be easily evaluated. When an unstable stream is ejected due to the lower viscosity of the delivery medium, the viscosity and stable stream of delivery medium can be optimized by adding another viscous material, thereby changing the concentration of the delivery medium itself or of the material affecting the degree of viscosity in the crystallization solution. The most efficient preliminary study of sample delivery involves determining the optimal conditions of stable and continuous stream for sample delivery using the sample injector under the experimental conditions (e.g., temperature, vacuum or ambient pressure, etc.) that will be used in the SX experiment.

Although a variety of delivery media have been developed and applied, it may not be possible to use the reported delivery media due to the occurrence of chemical reactions between the specific protein sample and the delivery medium or due to the physical damage made to the crystals during the mixing process. Therefore, further development of delivery materials is continuously required in order to improve the possibilities of carrying out SX research. In order to develop new delivery mediums for SX, the following should be considered: (i) the delivery material should be able to store the crystal sample for a long period of time in a stable manner; (ii) the delivery material should maintain a stable and continuous stream at low flow rates from the sample injector; (iii) injection stream characterization should be performed through chemical compatibility experiments for the crystallization solution; (iv) the injection stream should be available in a thin ID nozzle; (v) the background scattering generated from the delivery medium should be low. In particular, a very stable and continuous injection stream is required to provide reliable results when conducting time-resolved SFX (TR-SFX). In addition, low background scattering from the delivery medium and a stable stream with a thin ID nozzle to minimize background scattering are important and will contribute significantly to measuring weak anomalous signals for de novo phasing [55]. In TR-SFX using delivery media, on the other hand, the delivery medium should not be deformed by the pump source (e.g., laser, chemical reagent). This may have a temporary physical and chemical effect on the crystal sample and may result in a problematic structure due to an unwanted and unexpected source. Although a variety of delivery materials have been developed to date, until now, there have been no studies on the physical and chemical changes of the delivery materials by the pump source in pump-probe experiments. The effects of pump source on the delivery materials used in future TR-SFX experiment should also be studied.

## Abbreviations

SFX	serial femtosecond crystallography
SMX	serial millisecond crystallography
SX	serial crystallography
XFEL	X-ray free electron laser
LCP	lipidic cubic phase
ID	inner diameter
OD	outer diameter
HA	hyaluronic acid
HEC	hydroxyethyl cellulose
NaCMC	carboxymethyl cellulose sodium salt
F-127	pluronic F-127
PEO	poly(ethylene oxide)
PAM	polyacrylamide
